# Pancreaticoduodenectomy for pancreatic adenocarcinoma in a patient with situs inversus abdomenalis, a case report

**DOI:** 10.1016/j.ijscr.2022.107220

**Published:** 2022-05-18

**Authors:** Ahmed M.M. Elkoussy, Ahmed M.I. Taha, Ramy A. Hassan, Kirollos W. Nazeh

**Affiliations:** a HPB Surgery and Liver Transplantation Unit, Surgery Department, Al-Rajhi University Liver Hospital, Assiut University, Egypt; b Radiology Department, Assiut University Hospital, Assiut University, Egypt

**Keywords:** SIT, Situs Inversus Totalis, SIA, Situs Inversus Abdomenalis, CA 19-9, Cancer Antigen 19-9, MRCP, Magnetic Resonance Cholangiopancreatography, Situs inversus, Pancreatic adenocarcinoma, Pancreaticoduodenectomy

## Abstract

**Introduction and importance:**

Situs Inversus (SI) is a rare congenital condition in which the abdominal and thoracic organs are located in a mirror image of the normal position in the sagittal plane. Although this condition does not affect normal health or longevity, its recognition is very important for treating many diseases, particularly those requiring surgical intervention. The relationship between situs inversus and cancer remain inconspicuous.

**Case presentation:**

We report a 64-year old male with Situs Iinversus Abdominalis with Pancreatic Adenocarcinoma. Radiographic modalities were very important in preoperative assessment of the patient. The patient was managed by pyloric preserving pancreaticoduodenectomy. The patient received adjuvant chemotherapy and free of recurrence for one year after operation.

**Conclusion:**

Surgeons must recognize the complexity of operative intervention with respect to aberrant anatomy. The occurrence of Situs Inversus in a patient with pancreatic cancer must not deter the surgeon from sound oncologic principles of pancreatic surgery. Referral to these cases to tertiary level center is of utmost importance.

## Highlights

• Inversus Abdomenalis (SIA) which is least frequent of them (1 in 22000 of the general population)
• Situs inversus abdomenalis may be accopanied with malrotation of abdomenal viscera including pancreas , small and large bowel.
• Recognition of SIA by the new imaging modality MSCT and MRCP is very important for treating many diseases, particularly in surgery.
• Surgeons must recognize the complexity of operative intervention with respect to aberrant anatomy to do surgery on sound oncologic principles.

## 1 Introduction and importance

Situs Inversus is a short form of the Latin phrase “Situs inversus Viscerum” meaning “inverted position of the internal organs”, as first described by Marco Severino in 1643 [1]. Situs Inversus Totalis (SIT) is a rare congenital condition in which the abdominal and thoracic organs are located in a mirror image of the normal position in the sagittal plane. The incidence of this phenomenon is approximately 1 in 10,000 [2]. Although this condition does not affect normal health or longevity, its recognition is very important for treating many diseases, particularly those requiring surgical intervention [3]. If the anomaly does not involve all chest and abdominal organs it is termed are Situs Inversus Partialis. If the anomaly involve the heart only it is called dextrocardia which is more common than SIT. If the anomaly involves abdominal organs only It is called Situs Inversus Abdomenalis (SIA) which is least frequent of them (1 in 22,000 of the general population) [4]. Situs Ambiguous is subtype in which there is visceral malposition and dysmorphism associated with indeterminate atrial arrangement [5].

We herein report the anatomical challenges that we faced during performance of pancreaticoduodenectomy for management of pancreatic adenocarcinoma in a patient with SIA.

## 2 Case presentation

The case report was done according to the scare 2020 criteria [6]**.** A 64-year-old male patient presented to EL-Rajhy University Liver Hospital, Assiut, Egypt with jaundice of 4 weeks duration, pruritus, and recent weight loss. There are no other medical or psychological comorbidities. Both rug history and family history were negative. Laboratory data showed a pattern of obstructive jaundice (total bilirubin 6.1 mg/dL, direct bilirubin 4.9 mg/dL and alkaline phosphatase 483 units/L) with impaired clotting profile (prothrombin time 18.1 s, INR 1.4). All other laboratory data were with in normal including tumor markers (carcinoembryonic antigen and CA 19-9).

**Fig. 1:**
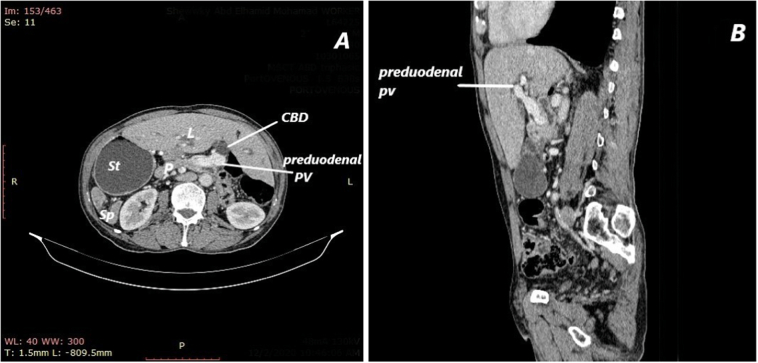
A: Computed tomography during portal phase shows heterotopic location of the liver, the stomach and the spleen The common bile duct is dilated. There are multiple small spleniculi, L: liver; St: stomach; Sp: spleen; P: pancreas. PV: portal vein. B: sagittal section showing preduodenal portal vein.

**Fig. 2:**
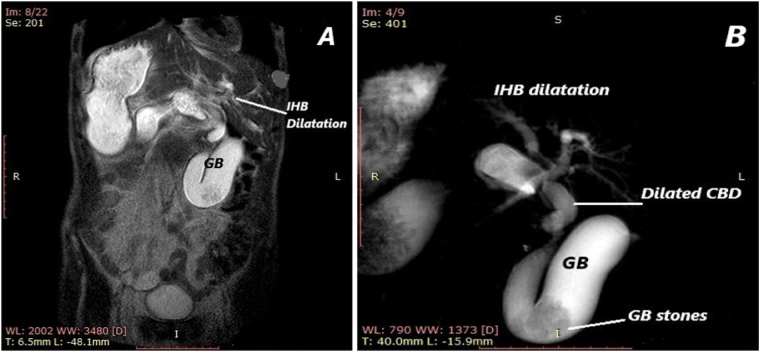
A: Magnetic resonance cholangiopancreatography (MRCP) Coronal T2WI showing distended gall bladder with dilated IHB channels. B: distended gall bladder with IHB channel dilatation, dilated CBD with abrupt end.

Imaging investigation in the form of abdominal ultrasonography, triphasic abdominal CT, Magnetic resonance cholangiopancreatography (MRCP) and chest CT revealed that the patient had SIA in the form of left sided liver, right sided spleen and stomach with normal left sided heart. The abdominal aorta and inferior vena cava were both normal sided. There was a 3.6 × 3 cm lesion in the head of a posteriorly located pancreas that was partially infiltrating a preduodenal portal vein with subsequent dilatation of biliary system and hugely dilated midline located gall bladder that contained a single large stone. There were small peripancreatic lymph nodes, no liver or distant metastasis (Figs. 1A,B, 2A,B). The management plan was discussed and consented by the patient.

Endoscopic Retrograde Cholangio-Pancreatography was done but cannulation of the ampulla of vater failed due to the anomalous location of the ampulla.

**Fig. 3:**
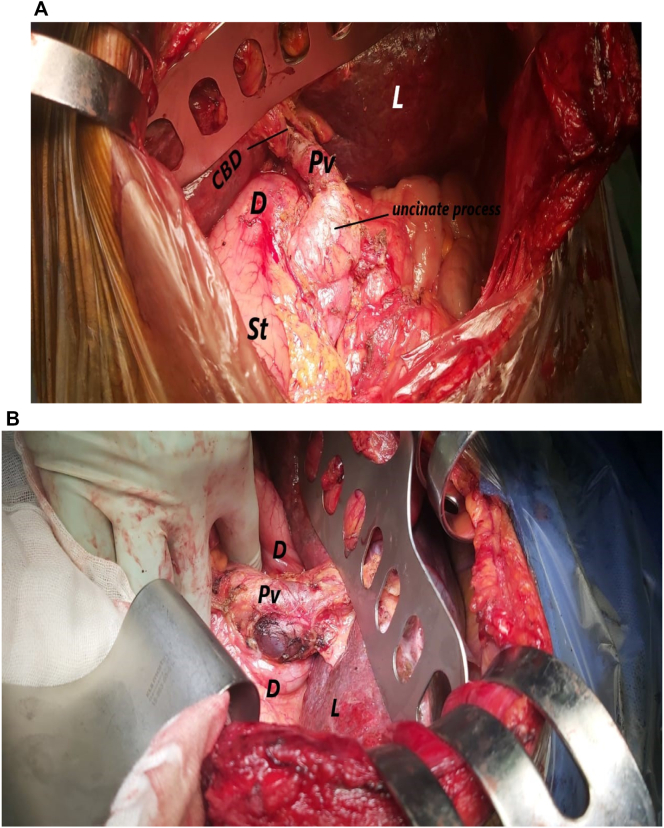
A: Shows heterotopic location of the liver and the stomach. There is a preduodenal portal vein and anteriorly sited uncinate process of the pancreas. B: The figure shows preduodenal portal vein. L: liver; St: stomach, PV: portal vein.

Pancreaticoduodenectomy was done. Laparotomy through a left subcostal incision with midline extension. The exploration confirmed SIA. The stomach fundus was on the right side with the pylorus, first and second parts of duodenum were extending from right to left to pass posterior to the portal vein. Then, the third and fourth parts of duodenum return to the right side and the jejunum starts at the right side. The rest of intestine showed non-rotation type of mal-rotation where the whole colon was in left side and the whole small intestine was in right side (Fig. 3A,B). The spleen was replaced by 4 separate spleniculi measuring 3 × 3 cm each in the right upper quadrant. The liver had minimal attachment to the retro peritoneum and could be easily brought to midline without mobilization. The gall bladder was hugely dilated and lying in midline. The body, neck and tail of pancreas were insinuated within the inverted C curve of the duodenum, all lying posterior to the portal vein. The uncinate process on the other hand was anteriorly located, anterior to the portal vein. The lesion was mainly uncinate process mass that was partially infiltrating portal vein. There was no liver or distant metastasis.

The anatomy of porta-hepatis was as follows:

The common bile duct extended cranially from an anterior location to the portal vein then becomes on its right side then pass behind the portal vein to join the posteriorly located duodenum. The portal vein was fusiformally dilated reaching around 3 cm without signs of portal hypertension. The hepatic artery was to the right of the portal vein and posterior to the bile duct. It was traced back to emerge independently from the normally located aorta. The operation started with Kocherization of the left sided duodenum started by its dissection from left kidney then continued in a left-to-right direction until the entire head of the pancreas was free of the aorta and superior mesenteric artery. Then opening of the lesser sac was done by the use of ligature to demonstrate the abnormal located pancreas posterior to the portal vein. Dissection of the pancreas from portal vein was done. Then dissection of the bile duct from the portal vein was done in spiral manner due to different relations of bile ducts to portal vein as previously prescribed. Partial portal vein resection was done at the end of mass resection. Classical pyloric preserving pancreaticoduodenectomy was done with portal vein resection and standard lymphadenectomy. The most difficult step was the transection of posteriorly located pancreas.

**Fig. 4:**
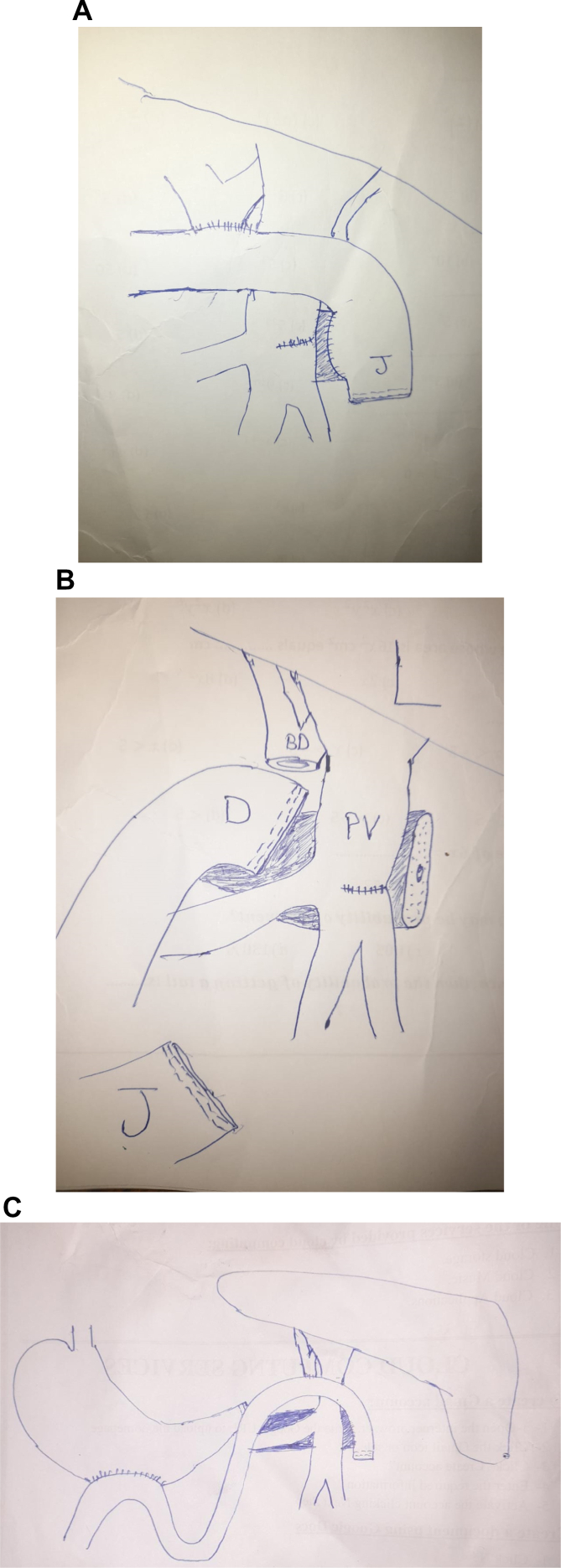
(A,B,C): Shows drawing for the reconstruction of the pancreas, bile duct and GiT after pancreaticodyodenectomy. P; pancreas, L: liver, J: jejunum, S: stomach, PV: Portal vein.

The reconstruction was challenging because of posteriorly located body of pancreas and malrotated jejunum. Duct to mucosa 4 layer pancreaticojejunstomy was done with a 4 fr silastic internal stent by 4/0 PDS. Hepaticojujonstomy was done. Duodenojujonstomy could not be done due to posteriorly located duodenum so its stump was closed by a stapler (The endo GIA™ Tri-staple ™ 60 × 3.5 mm blue cartridge, covidien) and gastrojejunstomy was done 40 cm distal to hepaticojejunstomy (Fig. 4A,B,C).

The specimen includes the pylorus, duodenum, part of jejunum, pancreatic head and uncinate process, gall bladder and cbd, part of the wall of portal vein. Ssplit cut section was whitish in colour obscuring pancreatic architecture and presence of gross cysts due to necrosis but there was no available photographs for the split cut of the tumor. The operation was done by the HBP surgery team in Elrajhy liver hospital under leadership of Dr. Ahmed MI Taha, Associate professor and consultant of HBP surgery.

The patient had an uneventful postoperative course. He stayed 2 days in ICU. He resumed oral feeding at postoperative day 2 in the inpatient word. The patient was monitored in the word clinical, laboratory radiological till removal of drains and discharge at day 7.after discharge, the patient was monitored in outpatient clinic weekly in the first month then monthly in the next six months.

**Fig. 5:**
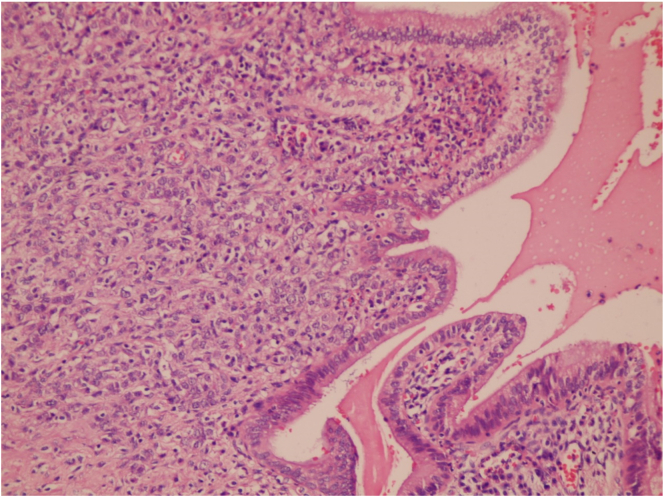
Microscopic examination of sections received from mass, stained by usual H & E stain, by 4 HPF, revealed poorly differentiated adenocarcinoma of pancreas infiltrating up to duodenal papillae.

A pathologic evaluation revealed a T3N1M0 poorly differentiated adenocarcinoma of the pancreas (Fig. 5). Safety margins were free with R0 resection of the tumour. The patient received adjuvant chemotherapy (GemCap) which is a combination of Gemcitabin and capecitabine for six months. The patient fellow up over the last 2 years revealed no recurrence.

## 3 Clinical discussion

Different cancers have been reported with SIT and SIA anomalies as cancer of stomach, colon, pancreas, biliary tract, ampulla of Vater, and kidney. However, they are not considered premalignant conditions. Despite their rarity and regarding the non-negligible number of published sporadic cases of cancers in these settings, a relationship between situs abnormalities and cancer has been suggested [7]. Sun et al. in 2017 reported all 79 previously published cases of malignancy with SIT, SIA and Situs Ambiguous which suggest the presence of relationship between cancer and these anomalies [8].

Interestingly, 25% of the cases with SIT or SIA have concurrent bronchiectasis and chronic sinusitis, and together these conditions are termed the triad of Kartagener syndrome. Our patient did not have Kartagener syndrome [9].

The first and the only case of periampullary cancer in association with SIA was reported by Malcolm M. Bilimoria et al., in 2001. It was for a 44-year-old female patient with SIA [10]. We here report the second case but in pancreatic carcinoma. Both cases had the same history of obstructive jaundice with raised levels of bilirubin. We did laparotomy through a left J-shaped incision but Bilimoria preferred a “Kocher” incision. Unlike Bilimoria, the preoperative biopsy in our patient by endoscopy was not done. Bilimoria identified no uncinate process but our patient had an anteriorly displaced uncinate process. We were not able to do duodenojejunostomy due to abnormal posteriorly sited duodenum. Also the pathology evaluation of Bilimoria case was ampullary origin with T2 N0 M0 but our case was pancreatic origin with T3 N1M0.

## 4 Conclusion

Surgeons must recognize the complexity of operative intervention with respect to aberrant anatomy. The imaging modalities like MSCT and MRCP before pancreatic resections are not very accurate because of the anatomic variations in patients with Situs Inversus. The occurrence of Situs Inversus in a patient with pancreatic cancer must not deter the surgeon from sound oncologic principles of pancreatic surgery. Referral to these cases to tertiary level center is of utmost importance.

## The guarantor of publication

Ahmed MI. Taha, MD

Email Address: Ahmed_Taha@Aun.Edu.Eg

Associate Professor And Consultant Of HBP Surgery, Assiut University, Egypt.

## Registry

None because This is the 2nd case reported with same condition.

The first and the only case of ampullary cancer in association with SIA was reported by Malcolm M. Bilimoria et al., in 2001.

## Consent for publication

Written informed consent was obtained from the patient for publication of this case report and accompanying images. A copy of written consent is available for review by the editor- In-chief of this journal on request.

## Ethical approval

The ethical committee approved the case report but waiting for official acceptance and number.

The Ethical Committee Of Faculty Of Medicine Assiut University, Egypt.

## Availability of data and material

The dataset used and analyzed during the current study is available upon request.

## Provenance and peer review

Not commissioned, externally peer reviewed.

## CRediT authorship contribution statement

Study Concept Or Design: Ahmed M. M. Elkoussy, Ahmed MI. Taha.

Data Collection: Ahmed M. M. Elkoussy, Kirollos W. Nazeh.

Data Analysis Or Interpretation: Ahmed M. M. Elkoussy, Ramy A. Hassan.

Writing The Paper: Ahmed M. M. Elkoussy, Ahmed MI. Taha, Ramy A. Hassan.

## Funding

None.

## Declaration of competing interest

None.
